# Plasma asymmetric dimethylarginine is associated with vulnerable plaque and long-term outcomes in stable coronary artery disease

**DOI:** 10.1038/s41598-023-32728-9

**Published:** 2023-05-09

**Authors:** Shao-Sung Huang, Wei-Chieh Huang, Chuan-Tsai Tsai, Ying-Ying Chen, Sheng-Hua Lee, Tse-Min Lu

**Affiliations:** 1 Division of Cardiology, Department of Internal Medicine, Taipei Veterans General Hospital, Taipei, Taiwan, ROC; 2 Department of Internal Medicine, School of Medicine, College of Medicine, National Yang Ming Chiao Tung University, Taipei, Taiwan, ROC; 3 Department of Biomedical Engineering, National Taiwan University, Taipei, Taiwan, ROC; 4 Division of Nephrology, Department of Internal Medicine, MacKay Memorial Hospital, Taipei, Taiwan, ROC; 5 Department of Health Care Center, Taipei Veterans General Hospital, Taipei, Taiwan, ROC

**Keywords:** Cardiology, Diseases

## Abstract

Asymmetric dimethylarginine (ADMA) is considered to be an atherogenic molecule. We aimed to investigate the relationship between ADMA and plaque vulnerability assessed by optical coherence tomography (OCT) in patients with stable coronary artery disease (CAD). Two hundred and forty-five patients with stable CAD undergoing OCT-guided percutaneous coronary intervention were included in this study and were divided into two groups according to their ADMA levels. Micro-vessel, macrophage accumulation, thin-cap fibroatheroma, intra-plaque calcium and lipid core content, and vulnerable score (VS) were evaluated by OCT analysis. The patients with higher ADMA levels had significantly higher calcium and lipid content (*p* < 0.001, respectively). There were significantly more micro-vessel and macrophage (32.8%, *p* = 0.004 and 52.5%, *p* < 0.001, respectively) and higher VS (87.7 ± 17.6, *p* < 0.001) in the higher ADMA group. Moreover, plasma ADMA level was significantly correlated with the intra-plaque lipid, calcium content and VS (*p* < 0.001, respectively). Plasma ADMA level was identified as an independent predictor of future adverse cardiovascular events, following OCT-guided PCI. In patients with stable CAD, higher plasma ADMA levels were significantly associated with the presence of intra-plaque lipid, calcification, vulnerable plaque, and poor long-term outcomes.

## Introduction

Impaired bioavailability of endothelium-derived nitric oxide (NO) and endothelial dysfunction may play a pivotal role in the initiation and progression of atherosclerosis^1^. As a well-characterized endogenous NO synthase inhibitor that can impair NO bioavailability and increase oxidative stress, asymmetric dimethylarginine (ADMA) has been suggested to be involved in the pathogenesis of endothelial dysfunction, microvascular dysfunction^2^ and atherosclerosis^3^. Moreover, elevation of ADMA level has been observed in patients with various atherosclerotic risk factors, including hypercholesterolemia, diabetes/insulin resistance, essential hypertension, and smoking^4,5^. Plasma ADMA levels have also been shown to be related to the intima-media thickness of the carotid artery^6^. Furthermore, accumulating evidence has also demonstrated that plasma ADMA levels might predict adverse cardiovascular (CV) events in patients with coronary artery disease (CAD)^7^. Although ADMA has been reported to be independently associated with the extent and functional severity of coronary atherosclerosis in patients with CAD^8^, the relationships between ADMA and atherosclerotic plaque characteristics, and vulnerability in patients with stable CAD have not been evaluated.

Currently, optical coherence tomography (OCT) with high resolution (10–20 µm) has been suggested to be a preferred imaging modality for detailed in vivo assessment of atherosclerotic plaques, and can provide information on plaque tissue composition, including intra-plaque lipid core and calcification^9^. In this study, we aimed to investigate the relationship between the tissue morphologic and compositional characteristics of coronary atherosclerotic plaque evaluated by OCT and the plasma ADMA levels in patients with stable CAD.

## Methods and materials

### Study design and objectives

From July 2016 to September 2018, 245 consecutive patients with non-invasive evidence of myocardial ischemia were enrolled and hospitalized. All patients received coronary angiography and OCT analysis was performed at the stenotic target lesion before coronary intervention. For patients with multi-vessel disease, the most severe coronary lesions were the culprit lesions and selected for OCT analysis. Clinical exclusion criteria included patients with left main disease and chronic total occlusion, acute coronary syndrome, acute decompensated congestive heart failure, acute and chronic infections, autoimmune diseases, malignancy with an expected life span of less than 1 year, unstable hemodynamic status, and inability to take dual antiplatelets therapy.

After angiographic and OCT analysis, all patients received OCT-guided PCI, which was defined as angiographically successful if residual stenosis was less than 30% and coronary Thrombolysis in Myocardial Infarction grade 3 flow was obtained at the end of the procedure without major complications. All patients had to take dual antiplatelets immediately after the procedure, specifically aspirin (100 mg per day) indefinitely and clopidogrel (300 mg loading dose and 75 mg maintenance per day) 12 months. Angiographic parameters, including minimal lumen diameter (mm), reference vessel diameter (mm), and percentage diameter stenosis, were obtained by quantitative coronary analysis (Cardiovascular Angiography Analysis System 8.5, Pie Medical Imaging B.V., Maastricht, the Netherlands). The study protocol was conducted according to the principles of the Declaration of Helsinki (1975) and was approved by the Institutional Review Board at Taipei-Veterans General Hospital. All participants provided written informed consent.

### OCT image acquisition and analysis^10^

OCT imaging was performed using the ILUMEN OPTIS™ system and Dragonfly™ (Abbott Vascular, Santa Clare, CA, USA) after an intracoronary injection of nitroglycerin. OCT images were analyzed by two independent experienced investigators blinded to the associated clinical information according to the Clinical Expert Consensus Document of OCT, and image analysis was performed offline using the ORW software (Abbott Vascular, Santa Clare, CA, USA). OCT imaging was assessed at 1 mm intervals and we selected the frame with the most severe stenosis for the OCT analysis. In all cases, OCT images of the entire length of the target lesion plus proximal and distal segments of 5 mm were included in the analysis^11^.

According to the OCT analysis, the atherosclerotic plaques were classified as fibrotic, fibrolipidic, or fibrocalcific types^12^. Detailed OCT definitions were given as: (1) Fibrotic type, with maximum lipid core arc ≤ 90° and maximum calcium arc ≤ 90°; (2) Fibrolipidic type, which was sub-classified as fibroatheroma, with maximum lipid core arc > 90° and minimal fibrous cap thickness > 65 µm, or thin-cap fibroatheroma (TCFA), with maximum lipid core arc > 90° and minimal fibrous cap thickness ≤ 65 µm at the thinnest part^11^; and (3) Fibrocalcific type, with maximum calcium arc > 90° and maximum lipid core arc ≤ 90°. Quantitative analysis for the content of calcium and lipid in the native vessels was performed at 1 mm intervals. We recorded the relative calcium index (RCI) = (mean calcium arc × calcium length) / (360 × analyzed length) and relative lipid core index (RLCI) = (mean lipid core arc × lipid core length) / (360 × analyzed length) as the relative volumetric measurement of intra-plaque calcium and lipid core content^12^. A calcified nodule was defined as an accumulation of multiple small protruding nodular calcifications with superficial thrombus or fibrin^13^. Macrophage accumulation was defined as a higher signal intensity within the plaque^11^, and a micro-vessel was defined as a circular black region with a diameter of 50–300 µm within the plaque^11^. Cholesterol crystals were defined as linear, highly backscattering structures within the plaque^11^. The vulnerable score (VS) was based on the presence of vulnerability markers on OCT images, such as TCFA, lipid pools, micro-vessels, and macrophage accumulation^14^. This score was calculated as the total number of all these vulnerability markers in 100 consecutive OCT frames (20-mm length)^15^.

### Laboratory measurements

Blood samples were collected after diagnostic angiography and immediately centrifuged at 3000 rpm for 10 min at 4 °C. Plasma samples were kept frozen at − 80 °C until analysis. Plasma ADMA level was measured by a competitive enzyme-linked immunosorbent assay kit (DLD Diagnostika GmbH, Hamburg, Germany) with a standard range from 0.1 to 5.0 μmol/L. The detection limit was 0.05 µmol/L. Estimated glomerular filtration rate (eGFR) was calculated according to the Modification of Diet in Renal Disease formula^16^.

### Clinical follow-up and outcomes

The clinical medical reports of enrolled patients were reviewed. Major adverse cardiovascular events (MACE) were a composite endpoint including CV death, non-fatal myocardial infarction (MI), and target vessel revascularization (TVR). CV death was defined as any death due to a definite CV cause or any death without clearly attributed to a non-CV cause. Non-fatal MI was known as significant new Q waves in at least two electrocardiography leads or an increase in creatinine kinase-MB fraction up to 3 times the upper limit of the reference range. TVR was defined as restenosis either within the target lesion or within the same epicardial coronary artery. A thrombus was defined as an irregular mass (diameter > 250 μm) attached to the luminal surface or floating within the lumen^10^.

### Statistical analysis

All continuous data are presented as mean ± standard deviation or with 95% confidence interval (CI). The differences of continuous data between two groups were compared by two-sample *t*-test while the differences among three or more groups were assessed by analysis of variance. Post-hoc comparisons were performed using the Bonferroni test. Categorical data between two groups were compared by means of a Chi-square test or Fisher’s exact test. Pearson’s correlation coefficients were calculated to examine possible correlations between continuous variables. Multivariate logistic/linear regression analyses were used to examine the association of plasma ADMA levels and RCI, RLCI and VS. Long-term MACE of both groups was estimated using the Kaplan–Meier method and were compared using the Log-rank test. A p-value of less than 0.05 was considered to be statistically significant. All statistical analyses were performed using SPSS statistical software (IBM SPSS Statistics for Windows, Version 22.0. Armonk, NY: IBM Corp.).

## Results

### Baseline characteristics of the study population (Table 1)

**Table 1:** Clinical characteristics and treatment in patients stratified by the median of asymmetric dimethylarginine.

Variables	ADMA			*p-*value
	All patients N = 245	≤ 0.9774 (µmol/l) N = 123	> 0.9774 (µmol/l) N = 122	
Baseline characteristics				
Age, (year)	66.5 ± 12.3	64.7 ± 11.5	68.2 ± 13.0	0.028
Male*, n (%)*	195 (79.6%)	101 (82.1%)	94 (77.0%)	0.325
Smoking*, n (%)*	122 (49.8%)	60 (50.4%)	62 (50.8%)	0.947
Hypertension*, n (%)*	184 (75.1%)	87 (70.7%)	97 (79.5%)	0.112
Diabetes*, n (%)*	120 (49.0%)	57 (46.3%)	63 (51.6%)	0.407
Atrial fibrillation*, n (%)*	22 (9.0%)	10 (8.1%)	12 (9.8%)	0.640
Hypercholesterolemia*, n (%)*	172 (70.2%)	87 (70.7%)	85 (68.7%)	0.856
CKD	36 (14.7%)	13 (10.6%)	23 (18.9%)	0.067
ESRD	19 (7.8%)	8 (6.5%)	11 (9.0%)	0.467
History of old MI*, n (%)*	39 (15.0%)	16 (23.0%)	23 (18.9%)	0.211
Old stroke*, n (%)*	24 (9.8%)	8 (6.5%)	16 (13.1%)	0.082
Medications				
Beta-blocker*, n (%)*	110 (44.9%)	53 (43.1%)	57 (46.7%)	0.568
ACEI/ARB*, n (%)*	220 (89.8%)	111 (90.2%)	109 (89.3%)	0.856
Statins*, n (%)*	150 (61.2%)	76 (61.8%)	74 (60.7%)	0.856
Laboratory measurements				
Total Cholesterol, (mg/dl)	157 ± 41.3	158 ± 44.2	154 ± 38.1	0.401
LDL-C, (mg/dl)	94.5 ± 34.6	95.3 ± 38.2	90.8 ± 30.9	0.320
Creatinine, (mg/dl)	1.63 ± 1.96	1.50 ± 1.90	1.76 ± 2.02	0.307
eGFR	45.2 ± 19.3	50.8 ± 14.6	41.1 ± 18.1	0.352
HbA1c	7.07 ± 1.42	7.01 ± 1.46	7.14 ± 1.39	0.401
ADMA, (μmol/l)	0.968 ± 0.241	0.783 ± 0.162	1.12 ± 0.15	< 0.001

ADMA, asymmetric dimethylarginine; ACEI, angiotensin converting enzyme inhibitor; ARB, angiotensin-II receptor blocker; CKD, chronic kidney disease; eGFR, estimated glomerular filtration rate; ESRD, end stage renal disease; LDL, low density lipoprotein; MI, myocardial infarction.

We enrolled 245 patients with symptomatic angiographically documented significantly stenotic coronary lesions in this study, and all patients received the OCT-guided interventional procedures. The mean age was 66.5 ± 12.3 years, and most of the patients were male (n = 195, 79.6%). Ninety-nine (40.4%) patients had multi-vessel disease, and the left anterior descending artery was the most evaluated vessel (n = 93, 38.0%). Nearly half of the patients had diabetes mellitus (DM) (n = 120, 49.0%) and 22.5% of the patients had chronic kidney disease (n = 36, 14.7%) or end stage renal disease (n = 19, 7.8%). The mean plasma ADMA level was 0.968 ± 0.241 µmol/L and the median level was 0.978 µmol/L. Significant negative correlations were observed between plasma ADMA level and eGFR (r =  − 0.260, *p* = 0.015). ADMA levels in DM patients were significantly higher than those of non-DM patients (1.00 ± 0.24 versus 0.932 ± 0.237, *p* = 0.019). We divided our study population into lower (n = 123) and higher ADMA level groups (n = 122) based on the median ADMA level. Patients in higher ADMA level were older (*p* = 0.028).

### OCT features of atherosclerotic plaques and ADMA

**Figure 1:**
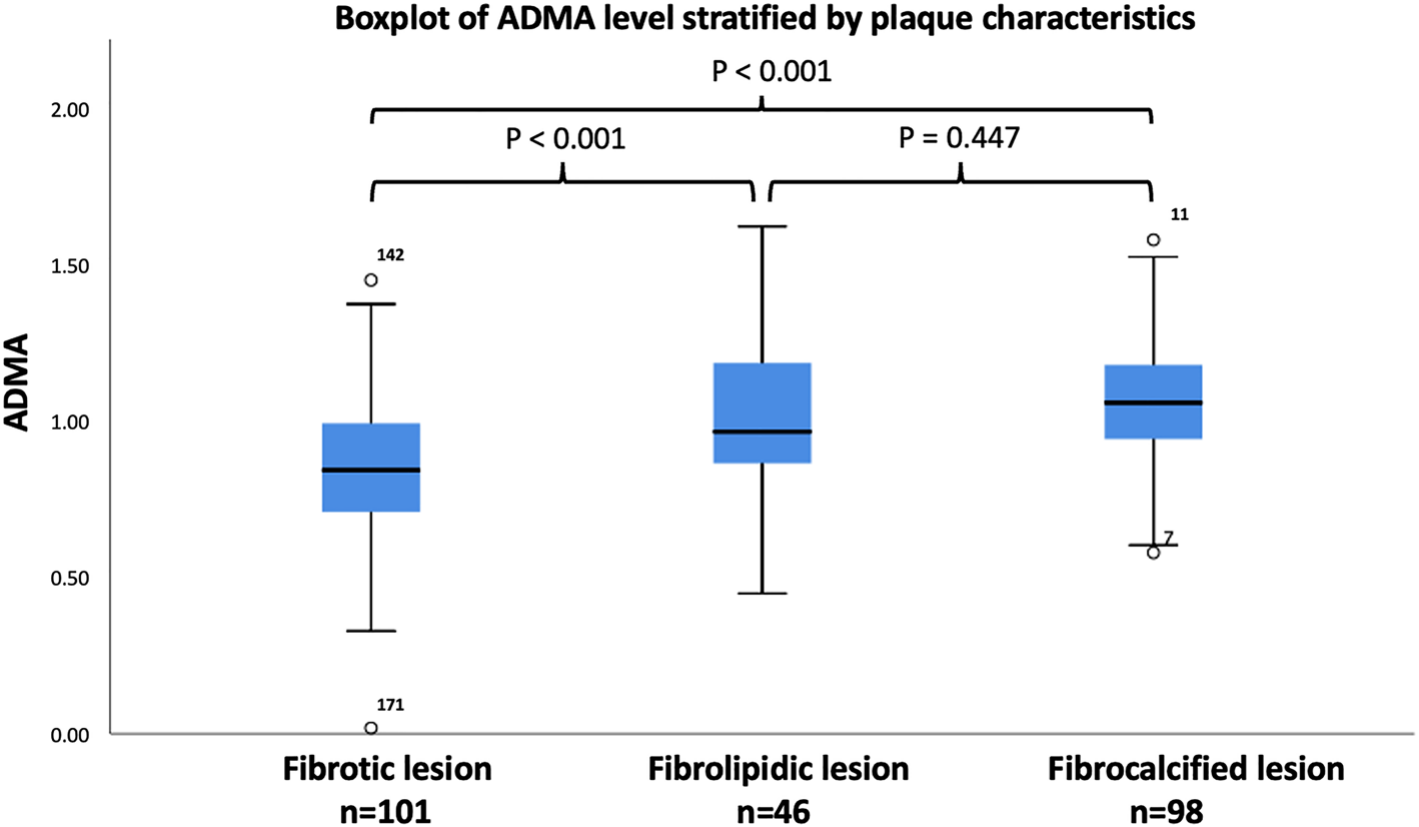
Boxplot of ADMA level was stratified by plaque characteristics and the ADMA level in fibrolipidic and fibrocalcified plaque are higher than those of fibrotic plaque.

**Figure 2:**
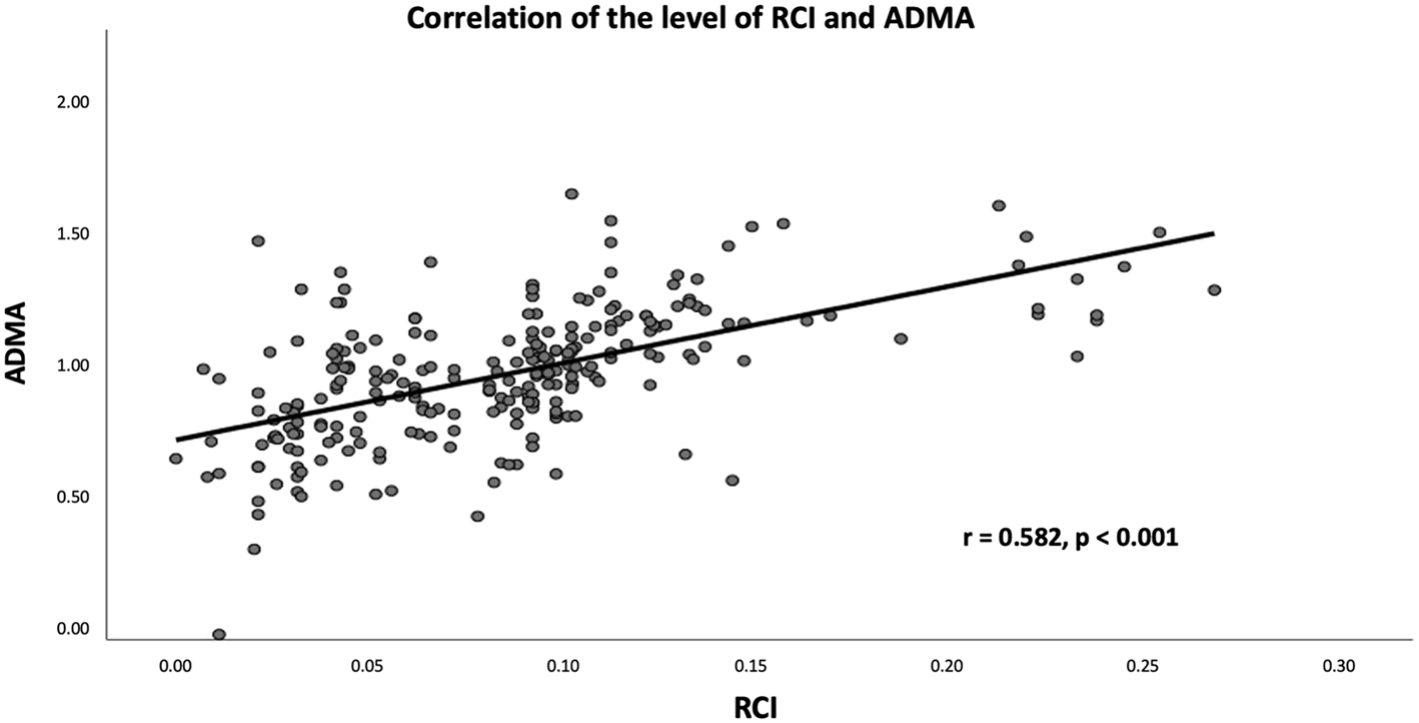
The correlation between ADMA level and RCI in all patients.

**Figure 3:**
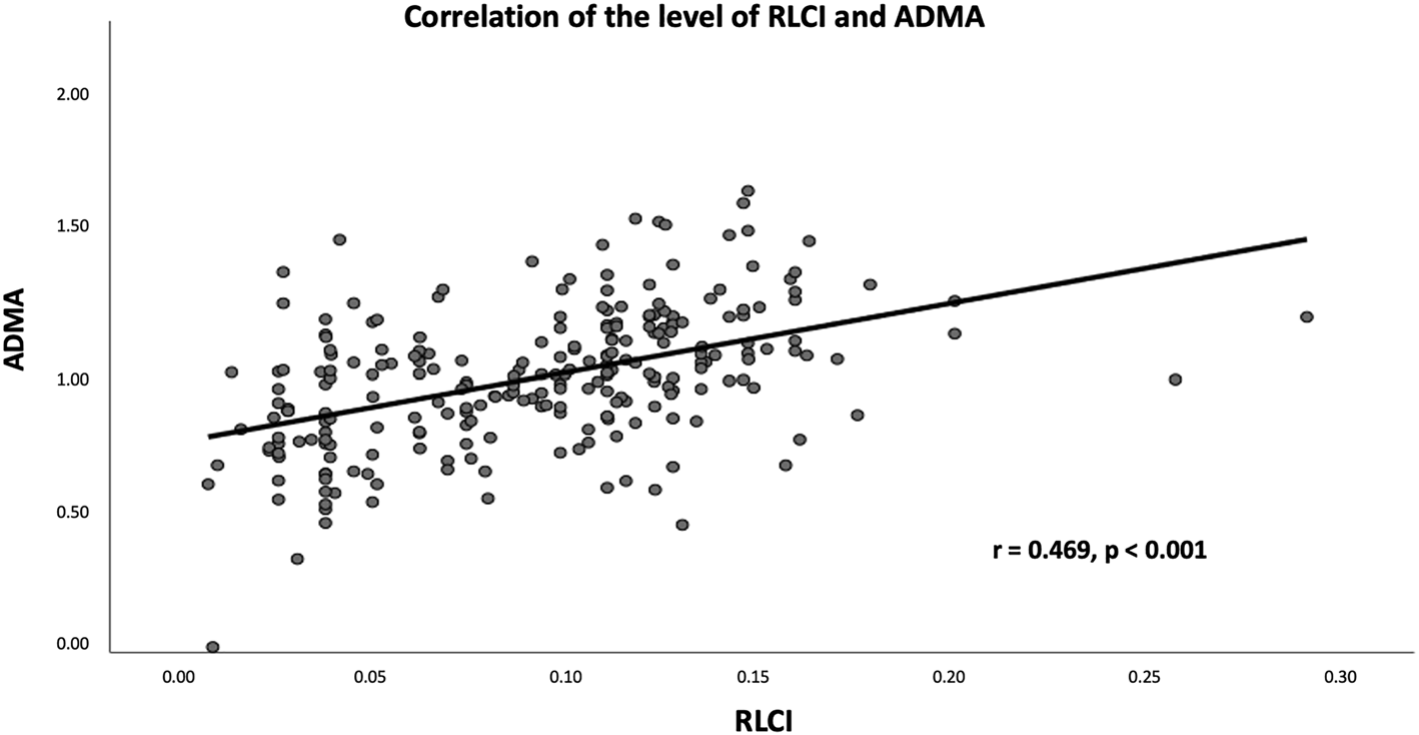
The correlation between ADMA level and RLCI in all patients.

**Figure 4:**
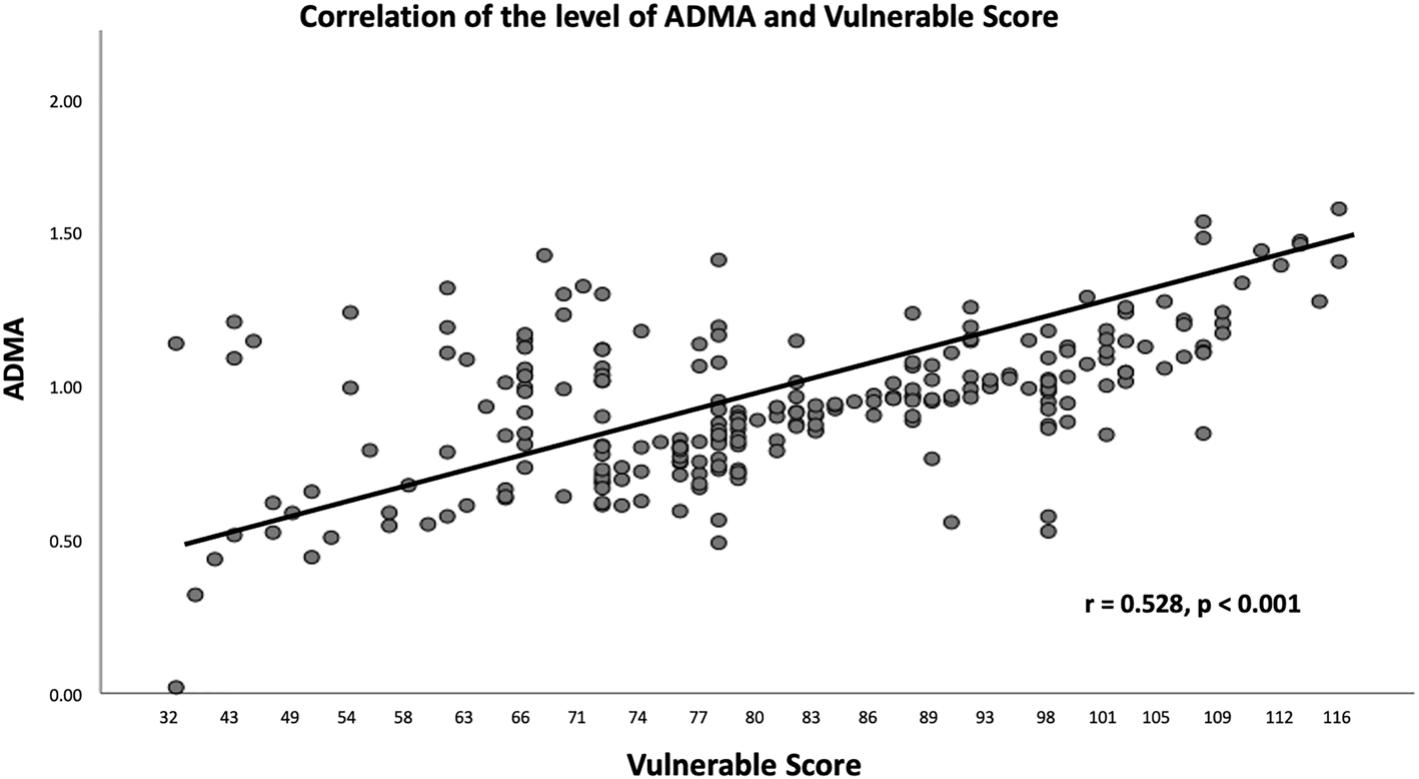
The correlation between ADMA level and vulnerable score in all patients.

The OCT characteristics are summarized in Table 2. According to the OCT findings, 101 (41.2%), 46 (18.8%), and 98 (40.0%) atherosclerotic lesions were classified as fibrotic, fibrolipidic, and fibrocalcific, respectively. Patients with fibrocalcific lesions had the highest ADMA level compared to those with fibrotic and fibrolipidic lesions (1.07 ± 0.20 versus 1.00 ± 0.23 versus 0.85 ± 0.23, Fig. 1). Patients with micro-vessels had higher ADMA levels than patients without (32.8% versus 17.1%, *p* < 0.001). Similarly, patients with macrophage accumulation had higher ADMA levels than patients without (52.5% versus 16.2%, *p* < 0.001). Thirteen TCFA (5.3%) and 10 calcium nodules (4.1%) were observed in the atherosclerotic lesions, neither of which reached a statistically significant difference between the groups. As for the lipid core (RLCI) and calcium content (RLCI) evaluated by OCT analysis, the RCI and RLCI were positively correlated with ADMA level in all patients (r = 0.582, *p* < 0.001; r = 0.469, *p* < 0.001, respectively, Figs. 2 and 3). Moreover, the mean VS of the target lesion from the OCT analysis was 81.4 ± 16.7 and was significantly correlated with the plasma ADMA level (r = 0.528, *p* < 0.001, Fig. 4). Target lesion in DM patients also had higher RCI, RLCI, and VS (*p* < 0.001, respectively). In the multivariate analysis adjusted for age, sex, and DM, plasma ADMA level remained a significant predictor for RCI (odds ratio [OR] = 0.116, 95% CI 0.033–0.054, *p* < 0.001), RLCI (OR = 0.068, 95% CI 0.051–0.086, *p* < 0.001), and VS (OR = 9.328, 95% CI 3.890–19.680, *p* < 0.001, Table 3).

**Table 2:** Angiographic characteristics and Optical coherence tomography characteristics of enrolled patients stratified by the level of ADMA.

Variables	ADMA			*p-*valve
	All patients N = 245	≤ 0.9774 (µmol/l) N = 123	> 0.9774 (µmol/l) N = 122	
Angiographic characteristics				
Diseased vessel number				0.941
SVD*, n (%)*	146 (59.6%)	74 (60.1%)	72 (58.7%)	
DVD*, n (%)*	71 (28.9%)	35 (28.6%)	36 (29.7%)	
TVD*, n (%)*	28 (11.4%)	14 (11.3%)	14 (11.6%)	
Target vessels				0.866
LAD*, n (%)*	93 (38.0%)	48 (39.0%)	45 (36.9%)	
LCX*, n (%)*	72 (29.4%)	35 (28.5%)	37 (30.3%)	
RCA*, n (%)*	80 (32.7%)	40 (32.5%)	40 (32.8%)	
Pre-PCI QCA				
MLD, (mm)	1.17 ± 0.14	1.16 ± 0.11	1.18 ± 0.12	0.966
RVD, (mm)	3.01 ± 0.48	3.00 ± 0.42	3.02 ± 0.51	0.944
DS, (%)	78.3 ± 12.2	74.3 ± 11.2	79.6 ± 16.2	0.823
Post-PCI QCA				
MLD, (mm)	3.01 ± 0.71	3.00 ± 0.78	3.02 ± 0.72	0.911
RVD, (mm)	3.02 ± 0.55	3.01 ± 0.48	3.02 ± 0.32	0.942
DS, (%)	11.2 ± 1.2	11.1 ± 1.1	11.2 ± 1.2	0.923
Type of Stents				0.962
BMS	51 (20.8%)	24 (19.5%)	27 (22.1%)	
DES	194 (79.2%)	99 (80.4%)	95 (77.9%)	
Stent length	21.6 ± 8.4	22.8 ± 8.2	23.9 ± 9.0	0.862
Stent diameter	3.02 ± 0.63	3.00 ± 0.66	3.03 ± 0.64	0.933
Rotational atherectomy	12 (4.90%)	3 (2.43%)	9 (7.37%)	0.002
Plaque characteristics				< 0.001
Fibrotic type*, n (%)*	101 (41.2%)	69 (56.1%)	32 (26.2%)	
Fibrolipidic type*, n (%)*	46 (18.8%)	24 (19.5%)	22 (18.3%)	
Fibrocalcified type*, n (%)*	98 (40.0%)	30 (24.4%)	68 (55.7%)	
TCFA*, n (%)*	13 (5.3%)	5 (4.1%)	8 (6.5%)	0.384
Calcium Nodule*, n (%)*	10 (4.1%)	4 (3.3%)	6 (4.9%)	0.550
Micro-vessel*, n (%)*	61 (24.9%)	21 (17.1%)	40 (32.8%)	0.004
Cholesterol crystal*, n (%)*	22 (9.0%)	10 (8.1%)	12 (9.8%)	0.561
Macrophage*, n (%)*	84 (34.9%)	20 (16.2%)	64 (52.5%)	< 0.001
Vulnerability score	81.4 ± 16.7	75.1 ± 13.2	87.7 ± 17.6	< 0.001
MLD, (mm)	1.13 ± 0.14	1.14 ± 0.10	1.12 ± 0.12	0.832
MLA, (mm^2^)	1.62 ± 0.51	1.58 ± 0.48	1.64 ± 0.31	0.852
Mean calcium arc, (degree)	49.2 ± 23.1	35.2 ± 13.5	63.4 ± 31.1	< 0.001
Mean lipid core arc, (degree)	45.6 ± 21.2	36.6 ± 11.6	61.3 ± 32.9	< 0.001
RCI	0.073 ± 0.048	0.062 ± 0.031	0.109 ± 0.053	< 0.001
RLCI	0.063 ± 0.038	0.070 ± 0.085	0.090 ± 0.036	0.021

BMS, bare metal stent; DES, drug eluting stent; DVD, double vessel disease; DS, diameter stenosis; LAD, left anterior descending artery; LCX, left circumflex artery; MLA, minimal lumen area; MLD, minimal lumen diameter; OCT, optical coherence tomography; QCA, qualitative comparative analysis; RCA, right coronary artery; RCI, relative calcium index; RLCI, relative lipid core index; RVD, reference vessel diameter; SVD, single vessel disease; TCFA, thin-cap fibroatheroma; TVD, triple vessel disease.

**Table 3:** The multivariate analysis of RCI and RLCI.

Variables	Relative calcium index (RCI)	
	Odds ratio (95% CI)	*p* value
Age	0.001 (0.000–0.002)	0.757
Sex	− 0.005 (− 0.010–0.042)	0.402
Diabetes	0.018 (− 0.032–0.008)	0.001
ADMA	0.116 (0.033–0.054)	< 0.001

Variables	Relative lipid core index (RLCI)	
	Odds ratio (95% CI)	*p* value
Age	0.001 (0.000–0.002)	0.516
Sex	− 0.006 (− 0.016–0.004)	0.263
Diabetes	0.026 (0.018–0.034)	< 0.001
ADMA	0.068 (0.051–0.086)	< 0.001

Variables	Vulnerability score	
	Odds ratio (95% CI)	*p* value
Age	1.291 (− 0.042–0.204)	0.198
Sex	2.160 (0.364–7.924)	0.032
Diabetes	9.743 (6.108–18.245)	< 0.001
ADMA	9.328 (3.890–19.680)	< 0.001

ADMA, asymmetric dimethylarginine.

### Clinical outcomes of enrolled patients

**Figure 5:**
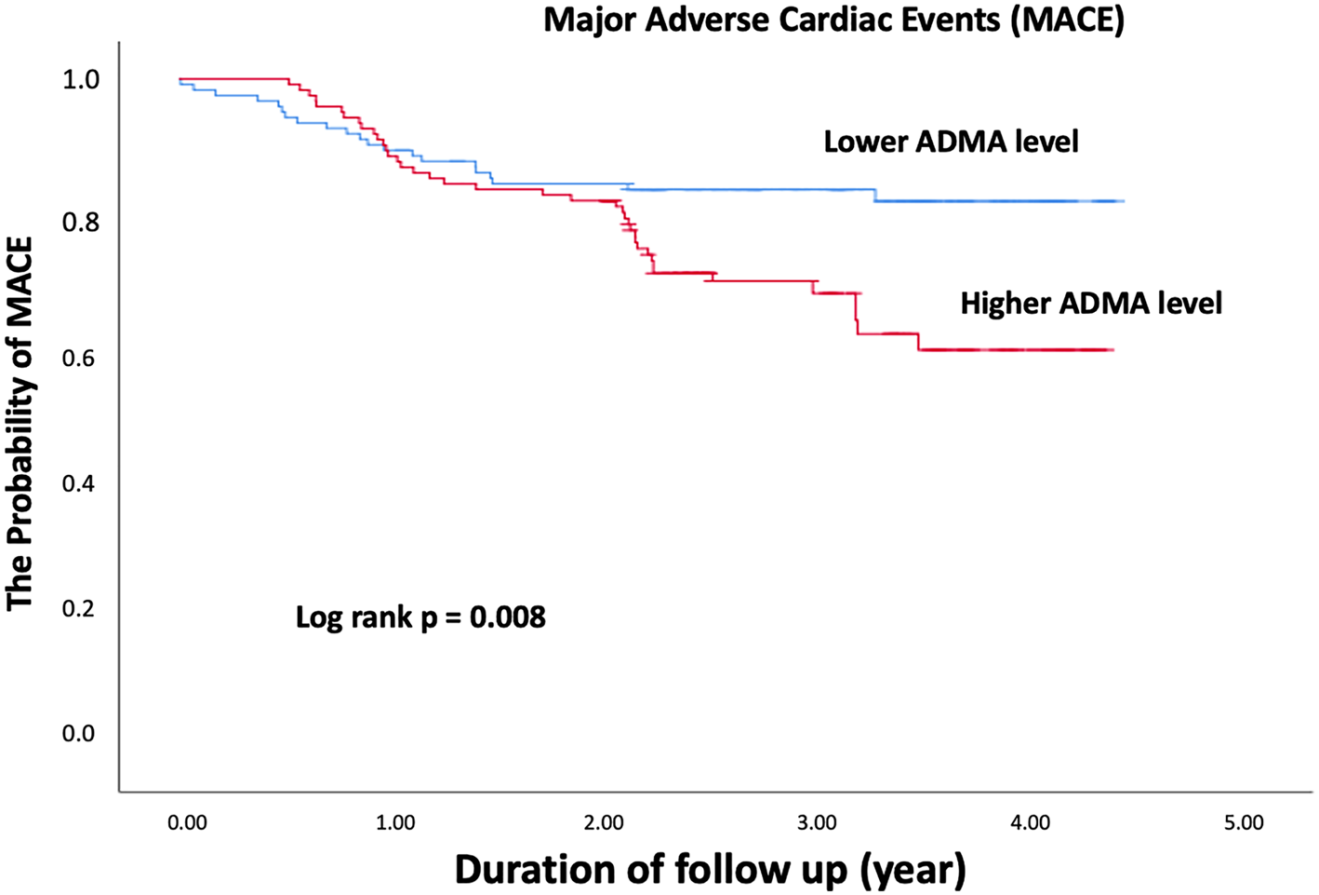
The Kaplan–Meier plane according to the low and high ADMA level, showing better long-term clinical outcomes in patients in lower ADMA group.

All patients were followed up completely for a mean period of 3.49 ± 1.12 years (median: 3.5 years, inter-quartile range: 2.68–4.37 years). During the follow-up period, 61 patients experienced MACE (24.9%), including 7 CV deaths (2.9%), 18 non-fatal MI (7.3%), 54 TVR (22.0%), 9 non-TVR (3.7%), and 4 stent thrombosis (1.6%). The patients in the higher ADMA group experienced more TVR (n = 35, 28.7%, *p* = 0.012) and MACE (n = 40, 32.8%, *p* = 0.004, Table 4). The Kaplan–Meier analysis showed better long-term clinical outcomes in the lower ADMA group (Fig. 5, Log rank *p* = 0.008). Furthermore, in the multivariate analysis adjusted for age, sex, and DM diagnosis, plasma ADMA level remained a significant predictor for long term MACE (*p* < 0.003).

**Table 4:** Clinical outcomes of enrolled patients depending on the level of ADMA.

Variables	ADMA			*p*-valve
	All patients N = 245	≤ 0.9774 (µmol/l) N = 123	> 0.9774 (µmol/l) N = 122	
CV death*, n (%)*	7 (2.9%)	4 (3.3%)	3 (2.5%)	0.709
Non fatal MI*, n (%)*	18 (7.3%)	7 (5.7%)	11 (9.0%)	0.319
TVR*, n (%)*	54 (22.0%)	19 (15.4%)	35 (28.7%)	0.012
Non TVR*, n (%)*	9 (3.7%)	3 (2.4%)	6 (4.9%)	0.288
Stent thrombosis	4 (1.6%)	2 (1.6%)	2 (1.6%)	0.998
Edge dissection	0	0	0	NA
MACE*, n (%)*	61 (24.9%)	21 (17.0%)	40 (32.8%)	0.004

ADMA, asymmetric dimethylarginine; CV, cardiovascular; MACE, major adverse cardiac events; MI, myocardial infarction; TVR, target vessel revascularization.

## Discussion

In this study, we found that in patients with stable CAD, the plasma ADMA level was significantly associated with the presence of intra-plaque calcification, lipid content, and presence of micro-vessels and macrophages, all features suggesting a complex and vulnerable plaque. Moreover, increased ADMA levels also independently predicted the future adverse CV outcomes after OCT-guided PCI.

ADMA, as an endogenous NO synthase inhibitor, has been considered to be an atherogenic molecule^4,17,18^. In the vascular endothelium of patients with CAD, ADMA is associated with decreased vascular NO bioavailability, higher systemic oxidative stress, increased vascular superoxide generation, and endothelial NO synthase uncoupling^19^. Notably, our previous study demonstrated that ADMA deregulates the cholesterol metabolism of macrophages and promotes the formation of foam cells, which are key events for the initiation and progression of atherosclerosis^20^. In this study, we first showed that plasma ADMA level was significantly associated with the lipid content of coronary atherosclerotic plaque, which is in line without previous findings, and taken together suggesting that ADMA might play an important role in the pathogenesis and progression of atherosclerosis^20,21^. In contrast, OCT-detected microstructures, including micro-vessels and macrophage accumulation, were strongly associated with plaque vulnerability^22,23^, and the VS provided semi-quantified assessment of plaque vulnerability. Intriguingly, our finding showed that ADMA was positively associated with not only OCT-detected complexity, but also plaque vulnerability. As previous study have showed that the presence of vulnerable plaque might be associated with increased risk of MACE^24,25^, our finding are in line with the results of subsequent group, which showed that the increased baseline ADMA concentration was independently associated with future cardiovascular events in 1874 patients with CAD^26^. Therefore, ADMA might be a risk factor of stable CAD, and the measurement of plasma ADMA level might have a potential implication in stable CAD. However, these finding needs to be confirmed in larger cohort.

Coronary artery calcification has been regarded as a surrogate for measuring the total atherosclerotic plaque burden and may predict future adverse CV events^27^. Extensive coronary artery calcification may be associated with reduced vascular compliance, abnormal vasomotor responses, and impaired myocardial perfusion^28^. Atherosclerotic plaque evolution, inflammation, and apoptosis of inflammatory cells may contribute to the initiation and progression of coronary artery calcification^29^. ADMA may be involved in these processes, as it has been reported to accelerate foam cell formation^18^ and induce apoptosis of endothelial cells by increasing oxidative stress^30^. Although few studies have evaluated the role of ADMA in coronary artery calcification, some may shed some light on their relationship. In the CARDIAC study, an independent relationship was found between plasma ADMA levels and the degree of coronary artery calcification detected by computer tomography^31^. These results together suggest that ADMA might be involved in the development of calcific atherosclerotic plaque, which is in line with our findings. As heavy coronary artery calcification may increase the risk of peri-procedure complications, procedure failure, and long-term outcomes, the close association of intra-plaque calcification and ADMA might be out of the contributing factor. This supports the observation that elevated plasma ADMA levels predict adverse CV events in patients with CAD undergoing PCI^7^.

Recently, one meta-analysis^32^ evaluated the prognostic value of blood ADMA level in patients with CAD and provided that evidence that elevated ADMA level is associated with an increased risk of all-cause mortality and MACEs in patients with CAD. Specifically, CAD patients with the highest ADMA level had approximately 2.1-fold higher risk of all-cause mortality, 2.49-fold higher risk of cardiovascular mortality, and 1.71-fold higher risk of MACEs. Their results suggest that ADMA level may serve as an important predictor of worse outcomes in CAD patients.

In this study, all patients received standard OCT-guided PCI, standard post-PCI care, such as modification of risk and medication. However, the outcomes were still noted after 2-year observation. It is interesting to address this question. Although angioplasty, stenting, and image-guided PCI, including OCT-guided PCI, may improve the long-term outcomes after PCI^33–35^, our results showed ADMA still remains a significant predictor for further adverse events after PCI. Therefore, it is key to understand the detailed mechanisms which underly plaque formation is key to the development of new treatments. These findings implicate ADMA as a culprit molecule in the development of atherosclerosis.

There are several limitations of our study. First, this was a single-center study with a limited sample size. Therefore, selection bias and potential confounding factors, such as medication, may exist. Medication affecting microvascular function will have impact on measurements of ADMA in patients. However, in this cohort, we collected blood samples based on our standard principal, which had applied to our previous publications^36,37^. Second, the effects of statin therapy on plasma levels of ADMA were reported in previous report and the findings showed a significant reduction in plasma ADMA concentrations. In this study, the population is constituted in any case of 60% of statin users and it will result in increased calcium content of plaques. However, the patients in our group received the standard post PCI care and modification of risks. Third, Intracoronary OCT appears to be feasible, and identified most architectural features detected by IVUS. OCT may provide additional detailed structural information. However, we acknowledged OCT is not the best technique for quantitative analysis of plaque features especially considering that each patient shows many plaques. Forth, the cross-sectional nature of our study precludes the cause-effect inferences about the links between ADMA and atherosclerotic plaque characteristics. Finally, this study was not a follow-up interventional study, and the relationship between plaque progression/regression and ADMA remains unclear. A follow-up study with a larger sample size is required to determine the predictive value of ADMA for plaque progression.

## Conclusion

In this study, plasma ADMA levels were significantly associated with the presence of intra-plaque calcification, lipid content, and plaque vulnerability in patients with stable CAD. Moreover, increased ADMA levels were also associated with worse long-term CV outcomes in patients undergoing OCT-guided PCI.

## Data Availability

The datasets used and/or analysed during the current study available from the corresponding author on reasonable request.
